# ASSOCIATION OF ALCOHOL CONSUMPTION WITH IMMUNE CELL PHENOTYPES IN THE FRAMINGHAM HEART STUDY

**DOI:** 10.1093/geroni/igae098.3799

**Published:** 2024-12-31

**Authors:** Ahmed Ragab, Jiachen Chen, Yumeng Cao, Margaret Doyle, Kathryn Lunetta, Joanne Murabito

**Affiliations:** Boston University, Boston, Massachusetts, United States; Boston University School of Public Health, Boston, Massachusetts, United States; Boston University, Boston, Massachusetts, United States; University of Vermont Larner College of Medicine, Colchester, Vermont, United States; Boston University, Boston, Massachusetts, United States; Chobanian and Avedisian School of Medicine, Boston University, Boston, Massachusetts, United States

## Abstract

Alcohol consumption, prevalent among 2.3 billion people worldwide, accounts for 4.7% of global mortality. While excessive drinking is harmful, moderate intake may have nuanced effects on immune function, though this relationship is not well-studied. We analyzed the relationship between average daily alcohol consumption and immune cell phenotypes among Framingham Heart Study Offspring participants. Consumption was examined in multiple ways: categorized and ordinalized (nondrinkers, moderate, at-risk, heavy), using average grams per day, and dichotomized as light vs heavy drinkers, with and without non-drinkers omitted. We profiled immune cell phenotypes using flow cytometry, including T, B, and NK cells, and T subtypes: CD4+, CD8+, CD4+CD28-CD27-, CD8+CD28-CD27-, CD4+ TEMRA, CD8+ TEMRA, CD4+/CD8+ ratio, CD4+(TN/TM), CD8+ (TN/TM), CD8+ Tc17/Treg ratio, CD4+ Th17/Treg ratio and GranzymeB CD8+/CD4+ratio. We used linear mixed effects models adjusting for age, sex, and cytomegalovirus status to test for associations, and FDR≤0.05 within an analysis set (defined by consumption definition) to declare significance. Among 928 participants (mean age 62 years, range 40-88, 52% female), 64% consumed alcohol. Compared to abstainers, CD4+(TN/TM) ratio was significantly increased in the moderate and at-risk daily consumption categories (β=0.26 for both, FDR=0.03). There were no significant linear associations between consumption and any of the immune cell phenotypes. However, among drinkers, we observed a significant negative association between average daily alcohol consumption and CD8+(TN/ TM) ratio (β= -0.14 for each higher category of consumption, FDR=0.03). The effects of consumption appeared stronger in males than females. Our findings suggest alcohol consumption may impact immune cell phenotypes.

